# Infective Endocarditis with Antineutrophil Cytoplasmic Antibody: Report of 13 Cases and Literature Review

**DOI:** 10.1371/journal.pone.0089777

**Published:** 2014-02-25

**Authors:** Chun-Mei Ying, Dong-Ting Yao, Hui-Hua Ding, Cheng-De Yang

**Affiliations:** Department of Rheumatology, Renji Hospital, Shanghai Jiaotong University School of Medicine, Shanghai, China; Royal College of Surgeons, Ireland

## Abstract

**Objective:**

Chronic infections tend to induce the production of antineutrophil cytoplasmic antibody (ANCA). Infective endocarditis (IE) has been reported to exhibit positive ANCA tests and to mimic ANCA-associated vasculitis, which may lead to a misdiagnosis and inappropriate treatment. The aim of this study was to clarify whether there is any difference in the clinical features between ANCA-positive IE and ANCA-negative IE.

**Methods:**

A retrospective study was carried out on 39 IE patients whose proteinase 3 (PR3)-ANCA and myeloperoxidase (MPO)-ANCA levels were measured. After dividing the patients into ANCA-positive and ANCA-negative IE, we compared their clinical features.

**Results:**

we compared 13 ANCA-positive IE patients with 26 ANCA-negative IE patients. All 13 ANCA-positive IE patients were proteinase-3-ANCA positive. Compared with the ANCA-negative IE group, the prevalence of edema of the lower extremities, the serum lactate dehydrogenase (LDH) level and positive blood cultures rate were higher in ANCA-positive IE group, but there was no significant difference in other clinical features.

**Conclusion:**

Therefore, if a patient presents with fever, arthralgia, skin rash and is ANCA-positive, appropriate steps should be taken to exclude infection (especially IE) before confirming the diagnosis of ANCA-associated vasculitis and embarking on long-term immunosuppressive therapy.

## Introduction

Antineutrophil cytoplasmic antibodies (ANCAs) directed against proteinase-3 (PR3) or myeloperoxidase (MPO) are important diagnostic markers for small small-vessel vasculitic syndromes (i.e. Granulomatosis with polyangiitis, microscopic polyangiitis, Eosinophilic granulomatosis and polyangiitis), which are commonly referred to as ANCA-associated vasculitis (AAV) [1]. However, several infectious diseases, particularly infective endocarditis (IE), have been reported to exhibit positive ANCA tests and to mimic AAV, which may lead to a misdiagnosis and inappropriate treatment [2]–[20]. Hence, IE is of particular importance in the differential diagnosis of AAV because the misdiagnosis of an infectious disease as AAV and the administration of immunosuppressive therapy could worsen the infection and lead to disastrous consequences.

In this report, we describe 13 patients with IE who had positive findings upon testing for ANCA by an antigen-specific enzyme-linked immunosorbent assay (ELISA). We then compared those findings with 26 ANCA-negative patients as well as cases reported in the literature.

## Methods

This study was approved by the Ethics Review Board of Shanghai Jiaotong University (Shanghai, China). All patients including the guardians on the behalf of the minors participants provided written informed consent to be included in the study.

### Patients

A total of 161 patients being treated at Shanghai Jiaotong University were diagnosed as having IE according to the modified Duke criteria [21] between January 2003 and June 2012. We have ruled out the patients with primary ANCA-associated-disease who occur super-infection or IE. Of the 161 patients with IE, only 39 individuals (21 males; age, 46.7±13.5 (range, 17–75) years) had been tested for ANCA; the decision to measure ANCA had been made by the referring physicians. The remaining 122 patients who did not undergo ANCA measurement were excluded from this study. We classified the 39 patients as “ANCA-positive IE” or “ANCA-negative IE” and compared their clinical features. Investigations to exclude the possibility of drugs inducing ANCA were carried out for all patients.

### Laboratory Tests

Levels of anti-PR3 and anti-MPO in serum were measured with an ELISA. The following laboratory data were recorded: White blood cell counts in blood (WBC), C-reactive protein (CRP), erythrocyte sedimentation rate (ESR), hemoglobin (HB), serum aspartate aminotransferase (AST), serum alanine aminotransferase (ALT), serum lactate dehydrogenase (LDH), γ-glutamyl transferase (GGT), serum creatinine, serum albumin, hematuria, proteinuria.

### Literature search

We undertook a MEDLINE (National Library of Medicine, Bethesda, MD, USA) literature review using certain keywords in different combinations: “infective endocarditis”, “IE”, “subacute bacterial endocarditis”, “SBE”, “anti-neutrophil cytoplasmic antibodies”, “ANCA”, “ systemic vasculitis”, “anti-proteinase 3”, “PR3”, “anti-myeloperoxidase”, “MPO”, “Wegener's granulomatosis”, “microscopic polyangiitis”, “Churg–Strauss syndrome”, and “cardiac”. We listed the results in tables describing the clinical features of IE with ANCA in our cases and cases from the literature.

### Statistical analyses

Statistical analyses were carried out using SPSS software (SPSS, Chicago, IL, USA). Descriptive statistics are represented as the mean ± standard deviation. Chi-square or Fisher's exact test were adopted to analyze all categorical variables. The Student's *t*-test was used to analyze continuous variables with a normal distribution. Survival curves were obtained using Kaplan-Meier's method. The significance was obtained by a log-rank test. Statistical significance was established at a two-tailed level of <5%.

## Results

### Prevalence of ANCA in IE patients with ANCA data

Of the 39 IE patients with ANCA data, 13 (13/39, 33.3%) patients (7 males) were ANCA-positive, the remaining 26 (26/39, 66.7%, 14 males) were negative for ANCA. The median age was 51.1±12.4 years among ANCA-positive IE patients, and 44.5±13.7 years among ANCA-negative IE patients. There was no significant difference in age or sex between the two groups. The baseline characteristics and predisposing conditions are shown in Table 1.

**Table 1:** Baseline characteristic of IE patients positive or negative for ANCA.

	ANCA-positive (n = 13)	ANCA-negative (n = 26)	*P*
Age (mean±SD	51.1±12.4	44.5±13.7	0.144
Male/female	7/6	14/12	0.632
Prosthetic valve IE	0	2(7.7)	0.439
Native valve IE	13(100)	24(92.3)	
Normal valve	0	3(12.5)	0.260
Native valve predisposition	13(100)	21(87.5)	
Rheumatic valve disease	1(7.7)	1(4.8)	0.626
Congenital heart disease	2(15.4)	1(4.8)	0.322
Valvular heart disease	10(76.9)	19(90.5)	0.274
Valve involvement	10(76.9)	21(80.8)	0.544
Mitral	4(40.0)	13(61.9)	0.224
Aortic	3(30.0)	4(19.0)	0.401
Tricuspid	0	0	–
Pulmonary	0	1(4.8)	0.677
Aortic plus mitral valves	3(30.0)	3(14.3)	0.284

Values in parentheses are percentages.

Table 2 shows the clinical characteristics of ANCA-positive IE and ANCA-negative IE subjects. Fever was the most common symptom: it was observed in all ANCA-positive IE and 22 (84.6%) of ANCA-negative IE cases. Anemia was observed in 9 (69.2%) ANCA-positive IE and 17 (65.4%) ANCA-negative IE subjects. Splenomegaly was noted in 5 (38.5%) ANCA-positive IE and 10 (38.5%) ANCA-negative IE patients. Nephropathy was noted in 4 (30.8%) ANCA-positive IE and 7 (26.9%) in ANCA-negative IE cases, whereas 5 (38.5%) ANCA-positive IE and 4 (15.4%) ANCA-negative IE individuals complained of arthralgia. Edema of the lower extremities occurred in 5 (38.4%) ANCA-positive IE and 2 (7.7%) ANCA-negative IE cases. Rash was found in 2 (15.4%) patients and 1 (7.7%) patient had cerebral infarction among ANCA-positive IE subjects, whereas 2 (7.7%) patients had a rash and 2 (7.7%) had cerebral infarction in the ANCA-negative IE group. Two (15.4%) ANCA-positive IE patients showed finger clubbing but Janeway lesions and Osler nodes were uncommon. ANCA-positive IE cases had a higher prevalence of edema of the lower extremities. However, there was no significant difference in other symptoms between the two groups.

**Table 2:** Clinical features of IE patients positive or negative for ANCA.

	ANCA-positive (n = 13)	ANCA-negative (n = 26)	*P*
Fever (≥38°C)	13(100)	22(84.6)	0.182
Anemia	9(69.2)	17(65.4)	0.553
Splenomegaly	5(38.5)	10(38.5)	0.639
Nephropathy	4(30.8)	7(26.9)	0.542
Arthralgia	5(38.5)	4(15.4)	0.115
Edema of lower extremities	5(38.5)	2(7.7)	0.030
Rash	2(15.4)	2(7.7)	0.407
Cerebral infarction	1(7.7)	2(7.7)	0.747
Finger clubbing	2(15.4)	0	0.105

Values in parentheses are percentages.

### Laboratory findings

All 13 ANCA-positive patients were PR3-ANCA-positive. Increase counts of WBC was presented in 10 (76.9%) ANCA-positive IE and 12 (46.2%) ANCA-negative IE cases. High values for C-reactive protein (CRP) were found in all patients. An elevated erythrocyte sedimentation rate (ESR) occurred in 11 (84.6%) ANCA-positive IE and 24 (92.3%) ANCA-negative IE cases, whereas a low concentration of hemoglobin (HB) was noted in 9 (69.2%) ANCA-positive IE and 17 (65.4%) ANCA-negative IE patients. The level of AST was higher in 4 (30.8%) ANCA-positive IE and 5 (19.2%) ANCA-negative IE patients, and ALT level increased in 2 (15.4%) ANCA-positive IE and 2 (7.7%) ANCA-negative IE patients. High level of LDH was detected in 11 (84.6%) ANCA-positive IE and 9 (34.6%) ANCA-negative IE patients, and the level of GGT ascended in 5 (38.5%) ANCA-positive IE patients and 12 (46.2%) ANCA-negative IE patients. One (7.7%) ANCA-positive IE and 2 (7.7%) ANCA-negative IE patients exhibited elevated level of serum creatinine. Eight (61.5%) ANCA-positive IE patients and 16 (61.5%) ANCA-negative IE patients appeared with low level of serum albumin. Seven of 12 (58.3%) ANCA-positive IE patients and 11 of 24 (45.8%) ANCA-negative IE patients had hematuria, whereas 4 of 12 (33.3%) ANCA-positive IE patients and 10 of 24 (41.7%) ANCA-negative IE patients presented with proteinuria. LDH levels were higher in ANCA-positive IE. There was no significant difference between factors in other laboratory results.

Blood cultures were conducted in 37 patients of 39 cases and a causative microorganism was identified in 9 ANCA-positive IE patients (69.2%) and 6 ANCA-negative IE patients (25%). The prevalence of positive blood cultures was significantly higher in ANCA-positive IE subjects. *Streptococcus* was the leading microorganism in both groups. There were 10 microorganisms isolated from ANCA-positive patients: 9 (90%) *Streptococcus* spp. and 1 (10%) *Enterococcus* spp. Seven microorganisms were isolated from ANCA-negative IE subjects: 5 *Streptococcus* spp. (71.4%), 1 *Enterococcus* spp. (14.3%) and 1 *Staphylococcus* spp. (14.3%) (Table 3).

**Table 3:** Laboratory results of IE patients positive or negative for ANCA.

	ANCA-positive (n = 13)	ANCA-negative (n = 26)	*P*
PR3-ANCA	13	–	
MPO-ANCA	0	–	
Raised WBC	10(76.9)	12(46.2)	0.067
Raised CRP (>8 mg/L)	13(100)	26(100)	–
Raised ESR (>20 mm/h)	11(84.6)	24(92.3)	0.407
Reduced HB (<110 g/L)	9(69.2)	17(65.4)	0.553
Raised AST (>40 U/L)	4(30.8)	5(19.2)	0.337
Raised ALT (>75 U/L)	2(15.4)	2(7.7)	0.407
Raised LDH (>240 U/L)	11(84.6)	9(34.6)	0.004
Raised GGT (>58 U/L)	5 (38.5)	12 (46.2)	0.457
Raised creatinine (>140 μmol/L)	1(7.7)	2(7.7)	0.747
Reduced albumin (<35 g/L)	8 (61.5)	16 (61.5)	0.633
Hematuria	7(58.3%,7/12)	11(45.8%,11/24)	0.362
Proteinuria	4(33.3%,4/12)	10(41.7%,10/24)	0.456
Microbiology			
Positive blood culture	9(69.2)	6(25,6/24)	0.007
Pathogen	10*	7*	
*Streptococcus* spp.	9(90.0)	5(71.4)	
*Enterococcus* spp.	1(10.0)	1(14.3)	
*Staphylococcus* spp.	0	1(14.3)	

*Two pathogens were isolated from the same blood sample.

Values in parentheses are percentages.WBC: white blood cell; CRP: C-reactive protein; ESR: erythrocyte sedimentation rate; HB: hemoglobin; AST: aspartate aminotransferase; ALT: alanine aminotransferase; LDH: lactate dehydrogenase; GGT: γ-glutamyl transferase.

### Outcomes

**Figure 1:**
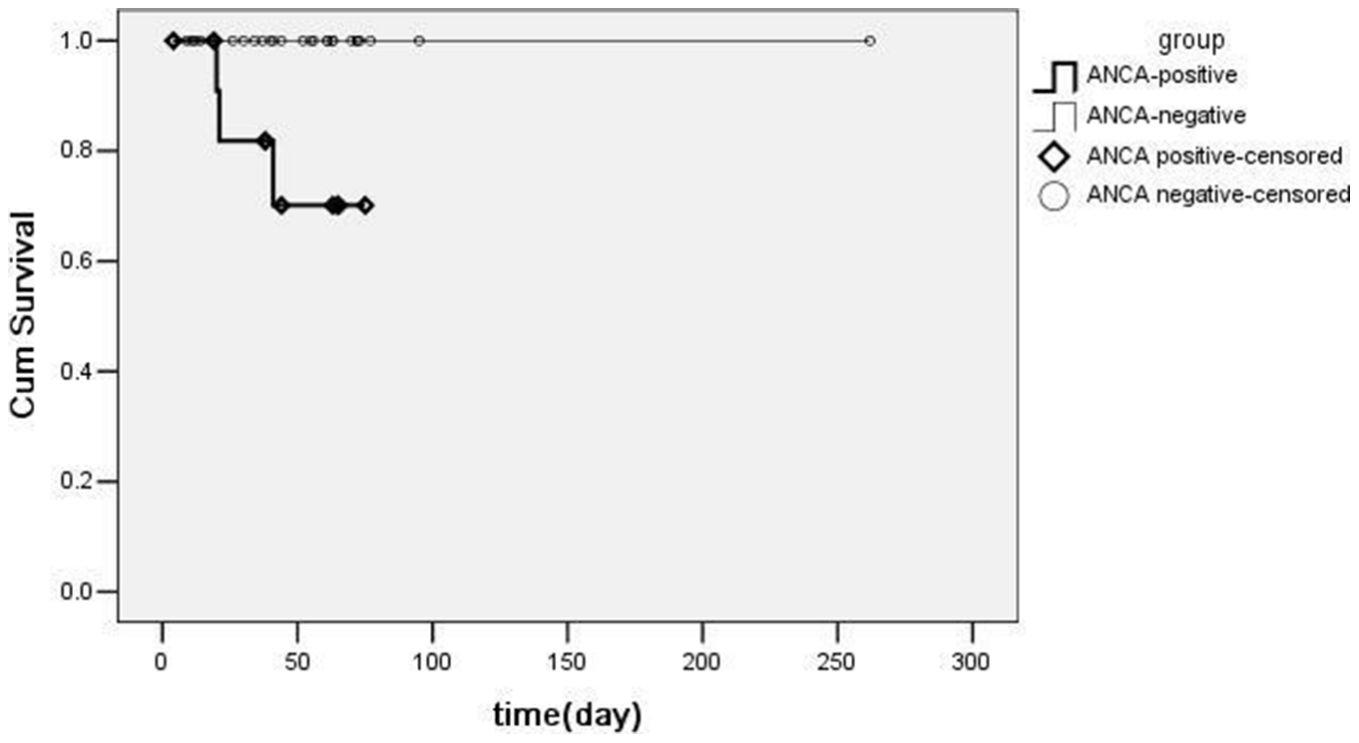
Kaplan-Meier's survival curves.

Overall, 3 patients in the ANCA-positive IE group died in hospital, and 1 patient was lost to follow-up. One patient died of renal failure and 2 patients died of acute heart failure. No patient died during the observation period in the ANCA-negative IE group and 2 patients were lost to follow up. The survival rate was significantly lower in ANCA-positive IE (*P* = 0.012, Fig. 1). All remaining patients survived and recovered after therapy in which 6 ANCA-positive IE patients and 17 ANCA-negative IE patients underwent heart operation, and there was no significant difference between the two groups (*P* = 0.566).

A Medline search of English-language articles describing IE patients with ANCA positive provided 31 reports of such cases. The characteristics of the 31 cases from the literature review are described in Table 4, along with the characteristics of the 13 patients from the current study.

**Table 4:** Characteristics of IE patients with ANCA: current and previous reports.

	Current report	Literature review [2]–[20]	*P*
N	13	31	
Age (mean±SD)	51.1±12.4	53.7±16.8	0.570
Male/female	7/6	26/5	0.046
Valve involvement	10/13	25/31	0.538
Mitral	4/10	8/25	0.470
Aortic	3/10	11/25	0.356
Tricuspid	0/10	3/25	0.351
Pulmonary	0/10	1/25	0.714
Aortic plus mitral	3/10	1/25	0.061
Mitral plus pulmonary and tricuspid	0/10	1/25	0.714
Clinical features			
Fever	13/13	24/31	0.069
Anemia	9/13	15/31	0.175
Splenomegaly	5/13	13/31	0.552
Nephropathy	4/13	20/31	0.043
Arthralgia	5/13	8/31	0.311
Edema of lower extremities	5/13	12/31	0.630
Rash	2/13	8/31	0.371
Purpura	0/13	7/31	0.069
Cerebral infarction	1/13	0/31	0.295
Finger clubbing	2/13	0/31	0.082
Laboratory results			
PR3	13/13	23/31	0.045
MPO	0/13	2/31	0.492
PR3+MPO	0/13	6/31	0.104
Hematuria	7/12	26/31	0.087
Proteinuria	4/12	19/31	0.095
Microbiology			
Positive blood culture	9/13	25/31	0.325
Pathogen	10	28	
*Streptococcus* spp.	9/10	16/28	0.063
*Enterococcus* spp.	1/10	4/28	0.604
*Staphylococcus* spp.	0/10	4/28	0.277
*Bartonella* spp.	0/10	2/28	0.538
*Neisseria* spp.	0/10	1/28	0.737
*Propionibacterium* spp.	0/10	1/28	0.737

## Discussion

Detection of ANCA is highly specific for the diagnosis of AAV (e.g., anti-PR3 antibody for Granulomatosis with polyangiitis). However, various infections can result in a positive ANCA test (especially IE). Usually, IE associated with ANCA is rare, but 31 cases have been reported [2]–[20]: 16 for *Streptococcus* spp., 4 for *Enterococcus* spp, 4, for *Bartonella* spp., 2 for *Staphylococcus* spp., 1 for *Neisseria* spp., 1 for *Propionibacterium* spp., and 6 culture-negative species. IE with ANCA can mimic the manifestations of AAV such as skin purpura and glomerulonephritis. Eight cases of ANCA-associated IE misdiagnosed as AAV have been published, including our report [7], [10]–[13], [15]. The differentiation between ANCA-associated IE and AAV may be difficult, but important differences in clinical presentation do exist between these entities. Chirinos et al. [12] described some pertinent differentiating features which may be more indicative of IE than AAV: organ involvement limited to skin and kidneys; positive blood cultures; abnormal levels of complement; immune deposits; and other autoantibodies (e.g., rheumatoid factor, antinuclear antibodies, cryoglobulins, anticardiolipin antibodies). AAV with valvular compromise involved almost exclusively the aortic valve and lead almost invariably to the need for valve replacement in the reported cases. Moreover, although discrete large vegetations seen on echocardiography suggest IE, small discrete vegetations have been rarely reported in AAV with valvula compromise. However, in their study age, sex, constitutional symptoms, ESRte and leukocytosis were not significantly different between the study groups. Bonaci-Nikolic et al. [15] compared 66 AAV patients with 17 PR3 and/or MPO-ANCA-positive patients with protracted infections, including 7 IE patients. They found that AAV patients more frequently had pulmonary and nervous system manifestations, while patients with infections more frequently had dual ANCA (high PR3, low MPO), aCL and anti-β2-GP I. Whereas there was no difference in frequency of lethality, renal failure and skin lesions in the two study groups. Haseyama, et al [4] suggested that in patients with high PR3 titers 50 U/ml in association with IE, it is initial to treated with immunosuppression. However, there have been 8 cases of IE with positive ANCA, including 2 cases in our report treated initially with immunosuppression or simultaneously with antibiotics because of delayed or equivocal diagnosis [7], [10], [11], [13], [15]. Four of these patients eventually died [10], [15]. Chirinos et al. [12] reckoned that histological examination of compromised valves revealed myxomatoid or fibroid degeneration of the valve tissue rather than granulomatous inflammation in most cases, explaining the lack of response to immunosuppressive therapy. Damage occurring during the initial valvulitis could lead to further valve distortion despite remission of the valvulitis with immunosuppressive therapy. Therefore, it is important that without convincing evidence of AAV and ruling out IE, there is no strong evidence to support immunosuppression.

In this retrospective study, 39 patients underwent testing for ANCA by ELISA, among which 13 (33.3%) were ANCA-positive. The decision to measure ANCA had been made by referring doctors as ANCA had not been measured consecutively in our hospital during the period in question. Even if we consider the selection bias of our data that ANCA levels were measured in only 39 cases among 161 inpatients in our hospital, the prevalence of ANCA in IE may be considered to be high. However, prospective studies are needed to clarify the precise prevalence of ANCA-positive IE. In addition, all 13 patients were PR3-ANCA-positive, a finding that is consistent with other reports. Of the 31 literature patients, 29 cases had PR3-ANCA, of which 6 cases had dual positive ANCA (PR3-ANCA and MPO-ANCA), whereas only 2 cases had MPO-ANCA. MPO-ANCA-positive IE, though rare, does exist.

All 13 PR3-ANCA associated patients had some manifestations mimicking AAV, such as fever, anemia, and splenomegaly. Streptococcal species were the most common pathogen, and cardiac-valve abnormalities were demonstrated in 10 patients, and all patients except 3 recovered with antibiotic therapy. We compared the clinical features of ANCA-positive IE patients and ANCA-negative IE patients: there was little difference between the two groups. These findings suggest that ANCA reflects the systemic inflammation rather than the activity of IE. As for laboratory findings, the white blood cell levels tended to be higher in ANCA-positive IE (*P* = 0.067), and LDH levels were higher in ANCA-positive IE. As LDH is a useful marker in activity of myocardial infarction, it is suggested that ANCA-positive IE was more active in cardiac lesions than ANCA-negative IE on admission. On the other hand, the rate of lower extremity edema was higher in ANCA-positive IE. We speculate that the edema was related to functional valve insufficiency. As this clinical feature is non-specific and the number of the patient was limited, their correlation needs more research. Interestingly, the prevalence of positive blood cultures was significantly higher in ANCA-positive patients than in ANCA-negative patients. This finding may suggest a link between infection and the development of ANCA. Negative blood cultures alone do not rule out the possibility of IE. Sugiyama et al. [14] reported a blood culture-negative IE patient who exhibited a positive ANCA test and simulated ANCA-associated vasculitis. Thus, when dealing with patients with negative blood cultures but positive ANCA tests, blood culture tests should be repeated.

Three ANCA-positive IE cases died but all ANCA-negative patients recovered, and the survival rate was lower in ANCA-positive IE in statistically. However, 2 of the 3 cases (50-year-old and 42-year-old women) were misdiagnosed as AAV and 1 (37-year-old man) exhibited serious congenital heart disease. Hence, the relationship between ANCA-positive IE status and survival needs further investigation.

To date, there was no any report descried the pathogenicity of ANCA in IE. We showed that there were few differences in clinical features between ANCA-positive and ANCA-negative IE patients. Further studies are necessary to clarify the role of ANCA in patients with IE. However, ANCA-positive IE can mimic AAV. If a patient has been found to be ANCA-positive and systemic vasculitis is suspected, physicians should take appropriate steps to exclude infection (especially IE) before planning and implementing long-term immunosuppressive therapy.
